# Actions and Adaptations Implemented for Maternal, Newborn and Child Health Service Provision During the Early Phase of the COVID-19 Pandemic in Lagos, Nigeria: Qualitative Study of Health Facility Leaders

**DOI:** 10.5334/aogh.3529

**Published:** 2022-02-21

**Authors:** Mobolanle Balogun, Aduragbemi Banke-Thomas, Uchenna Gwacham-Anisiobi, Victoria Yesufu, Osinachi Ubani, Bosede B. Afolabi

**Affiliations:** 1 Department of Community Health and Primary Care, College of Medicine of the University of Lagos, Lagos, Nigeria; 2 Department of Community Health, Lagos University Teaching Hospital, Lagos, Nigeria; 3 LSE Health, London School of Economics and Political Science, London, United Kingdom; 4 School of Human Sciences, University of Greenwich, London, United Kingdom; 5 Nuffield Department of Population Health, University of Oxford, Oxford, United Kingdom; 6 Department of Obstetrics and Gynaecology, College of Medicine of the University of Lagos, Lagos, Nigeria

## Abstract

**Background:**

The early phase of the COVID-19 pandemic led to significant disruptions in provision of maternal, newborn, and child health (MNCH) services, especially in low- and middle-income countries (LMICs) with fragile health systems, such as Nigeria. Measures taken to ‘flatten the curve’ such as lockdowns, curfews, travel restrictions, and suspension of public services inadvertently led to significant disruptions in provision of essential health services. In these countries, health facility leaders are directly responsible for driving changes needed for service delivery.

**Objective:**

To explore perspectives of health facility leaders in Lagos, Nigeria, on solutions and adaptations implemented to support MNCH service provision during the early phase of the COVID-19 pandemic.

**Methods:**

Key informant interviews were remotely conducted with purposively sampled 33 health facility leaders across primary, secondary, and tertiary public health facilities in Lagos between July and November 2020. Following verbatim transcription of recordings, data familiarization, and coding, thematic analysis was used to synthesize data.

**Results:**

Health facility leaders scaled down or discontinued outpatient MNCH services and elective surgeries. However, deliveries, newborn, immunization, and emergency services continued. Service provision was reorganized with long and staggered patient appointments, collapsing of wards and modification of health worker duty rosters. Some secondary and tertiary facilities leveraged technology like WhatsApp, webinars, and telemedicine to support service provision. Continuous capacity-building for health workers through training, motivation, psychological support, and atypical sourcing of PPE was instituted to be able to safely maintain service delivery.

**Conclusion:**

Health facility leaders led the frontline of the COVID-19 response. While they took to implementing global and national guidelines within their facilities, they also pushed innovative facility-driven adaptations to address the indirect effects of COVID-19. Insights gathered provide lessons to foster resilient LMIC health systems for MNCH service provision in a post-COVID-19 world.

## Introduction

The coronavirus disease (COVID-19) was declared a pandemic by the World Health Organization (WHO) on March 11, 2020. Since then, more than 190 million cases and over four million deaths have been recorded all over the world, as of July 27, 2021 [1]. Over the same period, in sub-Saharan Africa, Nigeria reported over 170,000 cases with 2,000 plus deaths [2].

At the beginning of the pandemic, response to the pandemic included lockdowns, curfews, travel restrictions, suspension of several public services all in a bid to ‘flatten the curve’. This response, in addition to the direct effect of the pandemic itself have led to significant disruptions in provision of essential health services. Of all services, maternal, newborn and child health (MNCH) services typically feel the brunt the most, as in previous infectious disease outbreaks. For example, with the 2014–15 Ebola virus disease outbreak in West Africa, an 80% reduction in maternal service delivery and an overall increase in maternal morbidity and mortality were reported [3]. With the COVID-19 pandemic, a study published before most countries went into lockdown estimated an increase of 8.3 to 38.6% in maternal deaths per month in 118 low- and middle-income countries (LMICs) including many in sub-Saharan Africa [4]. Evidence gathered thus far demonstrates that indeed there have been significant increase in stillbirths and maternal deaths in LMICs [5] There have also been severe disruptions in MNCH service provision and utilisation [6,7]. For example, one article reported reduction in antenatal care (ANC), family planning, and immunization in Bangladesh, South Africa, and Nigeria [8]. Reductions in access to and utilisation of essential MNCH services this crisis period can undermine progress towards the 2030 global consensus targets of reducing maternal deaths to less than 70 per 100,000 live births, neonatal mortality to at least as low as 12 per 1,000 live births and under-five mortality to at least as low as 25 per 1,000 live births [9].

To optimise provision of MNCH services during the pandemic, guidelines were developed at a global level and adapted for use at local levels [10,11]. Skilled health personnel including nurses, midwives, and doctors were at the forefront of actioning these guidelines [6]. In Lagos State, a survey amongst skilled health personnel, more than a third reported that maternal and newborn health services were not available at some time between March and June 2020, nine in ten skilled health personnel felt stressed working during this period, almost all skilled health personnel were concerned about the availability of personal protective equipment (PPE) and related guidelines, and only about 12% were satisfied with the preparedness of their health facilities for service provision [12].

Health facility leaders were responsible for many of the decisions taken to optimise health facilities for service provision during the COVID-19 pandemic. Despite a call to action made by researchers on the need to document and share solutions and adaptations [13], no study has been published that captured these efforts in an LMIC setting till date. Our objective in this study was to explore the perspectives of health facility leaders in Lagos, Nigeria, on solutions and adaptations implemented to support MNCH service provision during the early phase of the COVID-19 pandemic. The study was nested within a broader mixed methods study that assessed the impact of the COVID-19 pandemic on routine maternal, newborn, and child health services at all levels of care in Lagos State.

## Methods

### Setting

Lagos State is the economic nerve centre of Nigeria. With a population of 21 million people, it is the most populous state in Nigeria. The State has also been the epicentre of the COVID-19 pandemic in the country with over 22,000 laboratory-confirmed cases and 220 deaths, making up 14% and 11% of national figures respectively [14]. The lockdown in Lagos lasted for 108 days (complete lockdown: March 30–May 3, 2020 [35 days] and gradual easing lockdown: May 5–July 15, 2020 [73 days]) [15]. The different phases of the lockdown entailed limitation of local and interstate travel, public gathering, opening of non-essential businesses, and curfews as guided by the State’s Emergency Response Committee. As a result of the high number of COVID-19 cases in the State, a lot of resources were diverted towards management of the COVID-19 response [16].

Pre-pandemic, the State was implementing strategic plans in response to some sub-optimal indicators of MNCH including 76% facility-based delivery, 73% of women with postnatal check within two days of birth, maternal mortality ratio of 555 per 100,000 live births, neonatal mortality rate of 29 per 1,000 live births, and under-five mortality rate of 50 per 1000 live births [17,18]. In Lagos State, public sector health service provision is tiered (primary, secondary, and tertiary). There are 329 primary healthcare centres (PHCs) at the primary level, 27 general hospitals at the secondary level and five tertiary healthcare facilities (including two teaching hospitals and one federal medical centre that provide MNCH services) [19]. The state has 57 councils under five administrative zones with 38 to 145 PHCs and two to nine general hospitals per zone. Although concerns relating to unresponsiveness of service provision have been highlighted, women using MNCH services in Lagos public hospitals have reported that they were satisfied with the competency of health personnel and equipment in the higher-level facilities [20].

### Recruitment of participants

Health facility leaders who were responsible for operations in public hospitals and PHCs across the State were invited to partake in the study via phone calls. At the primary health care level, we targeted medical officers of health, apex nurses, and apex community health officers across five councils with a total of 39 PHCs under their supervision. At the secondary level, we recruited medical directors and heads of department/units providing MNCH services in five general hospitals. To get a good spread, the councils with their corresponding PHCs and the general hospitals were selected from the five administrative zones in the state. At the tertiary level, we used the two teaching hospitals providing MNCH services and targeted Chairmen Medical Advisory Committees (CMACs) and heads of departments/units.

### Data collection

Key informant interviews (KIIs) were conducted with health facility leaders between July and November 2020. This was the period after the gradual easing of the lockdown in the state [15]. A predesigned standard operating protocol was used to guide the process of data collection. The KIIs were conducted remotely via Zoom (Zoom Video Communications, San Jose, California, United States) by the principal investigator (MB). All the KIIs were audio-recorded and lasted between 32–47 minutes. Reflective notes were taken to supplement transcripts. Piloted topic guides were used to collect data. These topic guides included several open-ended questions that focused on experience and challenges of interviewees in leading service provision during the pandemic. During the KIIs, as expected of robust qualitative research, trustworthiness of the research was a focus [21]. The interviewer made efforts to establish rapport with the interviewees and verification of assertions of interviewees was done to ensure an accurate understanding had been captured by the interviewer. Data collection continued until data saturation was reached.

### Data analysis

Audio recordings from the KIIs were transcribed verbatim. A thematic analysis was conducted using Braun and Clarke’s six steps for thematic analysis: becoming familiar with the data, generating initial codes, searching for themes, reviewing themes, defining, and naming themes, and producing the report [22]. An inductive approach was taken in generating the codes. Open coding was conducted to ensure that no relevant information of the data was missed, in line with the exploratory approach taken for this analysis. Analysis was performed with the aid of computer-assisted qualitative data analysis software, NVivo 10 (QSR International, Memphis, Tennessee, USA). Illustrative quotes were extracted from the transcripts to reflect the core message within the key emerging themes.

### Ethical considerations

Ethical approval was received from the Health Research and Ethics Committee of Lagos University Teaching Hospital (LUTHHREC/EREV/0620/64). Social approval was obtained from the Lagos State Ministry of Health and permission to access health facility leaders was obtained from the Lagos State Health Service Commission, the Lagos State Primary Health Care Board, and the heads of facilities. Participation in the study was entirely voluntary. A waiver of signed informed consent was obtained from the ethics committee as the research was deemed to present minimal risk of harm to interviewees. Instead, verbal informed consent was obtained from the interviewees as well as consent for audio recording. The audio recordings were saved in a password-protected laptop and deleted after transcriptions after completed. Confidentiality of interviewees was maintained by not using identifiers. No financial incentive was offered.

## Results

In all, 33 health facility leaders were interviewed including one community health officer, nine nurses, and 23 doctors. Amongst the nurses, there were five nurse managers called ‘apex nurses’, three chief nursing officers, and one assistant director of nursing services. Amongst the doctors, there were ten heads of department/unit, five medical directors, five medical officers of health, two chairmen of Medical Advisory Committees, and one Director Clinical Services and Training. Twenty-two of the health facility leaders were female and 11 were male. All had between two months and 18 years of experience working as managers in their health facilities. Ten were leading health facilities based in rural areas and 23 in urban areas, ten were managers in PHCs, nine in general hospitals, and 14 in teaching hospitals (***Table 1***).

**Table 1:** Individual characteristics of respondents.


PARTICIPANT	SEX	ROLE	HEALTH FACILITY TYPE	URBAN/RURAL	YEARS IN ROLE

P1	Female	HOD Paediatrics	Tertiary	Urban	6 months

P2	Male	Medical Officer of Health	PHC	Rural	3 years

P3	Female	Apex Nurse	Secondary	Rural	12 years

P4	Female	Apex Nurse	PHC	Rural	4 years

P5	Female	Apex Nurse	PHC	Urban	2 years

P6	Female	HOD Nursing	Tertiary	Urban	4 years

P7	Male	HOD Obstetrics & Gynaecology	Tertiary	Urban	6 months

P8	Male	HOD ART	Tertiary	Urban	16 years

P9	Female	HOD Nursing	Tertiary	Urban	3 years

P10	Female	HOD Obstetrics & Gynaecology	Tertiary	Urban	2 years

P11	Female	Medical Director	Secondary	Rural	2 months

P12	Female	Medical Director	Secondary	Rural	3 months

P13	Female	Medical Director	Secondary	Urban	10 months

P14	Male	Medical Director	Secondary	Urban	3 years

P15	Male	Medical Officer of Health	PHC	Rural	1 year

P16	Female	Medical Officer of Health	PHC	Urban	4 years

P17	Female	Medical Officer of Health	PHC	Urban	12 years

P18	Male	HOD PMTCT	Tertiary	Urban	17 years

P19	Female	DCST	Secondary	Urban	9 months

P20	Female	Apex Nurse	Secondary	Urban	3 years

P21	Female	Apex Community Health Officer	PHC	Rural	4 years

P22	Female	Apex Nurse	Secondary	Rural	4 years

P23	Male	CMAC	Tertiary	Urban	2 years

P24	Male	HOD Community Health	Tertiary	Urban	1 year

P25	Female	HOD Paediatrics	Tertiary	Urban	2 years

P26	Female	Officer in Charge	PHC	Rural	2 years

P27	Female	Medical Officer of Health	PHC	Urban	10 years

P28	Female	HOU Neonatology	Tertiary	Urban	10 years

P29	Male	CMAC	Tertiary	Urban	2 years

P30	Female	Head Child ART	Tertiary	Urban	18 years

P31	Male	HOD Community Health	Tertiary	Urban	7 months

P32	Male	Medical Director	Secondary	Urban	1½ years

P33	Female	Apex Nurse	PHC	Urban	2 years


HOD/HOU – Head of department/Unit; DCST: Director Clinical Training and Services; CMAC: Chairman Medical Advisory Committee; ART: Anti-Retroviral Therapy program.

There were five key emerging themes. These are described in detail below:

*Theme 1: Scaling down and discontinuing certain MNCH service delivery*

Health facility leaders in secondary and tertiary facilities reported that the pandemic led to scaling down of outpatient health services, even before the nationwide lockdown. In addition, elective surgeries were postponed (***Table 2***; P13, urban tertiary facility; P32, urban secondary facility). However, emergency, childbirth, neonatal, and immunization (in primary and tertiary facilities) services continued to be provided (***Table 2***; P23, urban tertiary facility; P13, urban secondary facility]. To reduce intra-facility transmission, fewer skilled health personnel including nurses and doctors were allowed to come to work. While specific dates of discontinuing or reopening services varied across hospitals, most services appeared to have returned to normal for many after lockdown was eased in May 2020.

**Table 2:** Illustrative quotes for theme 1.


THEME 1 – SCALING DOWN AND DISCONTINUING CERTAIN MNCH SERVICE DELIVERY

“Before the lockdown, we scaled down activities at the clinic. We stopped regular outpatient clinic and attended mainly to emergencies. This affected both obstetric and gynaecological practices. During the lockdown, the same thing prevailed, and we are only attending to emergencies, no regular clinic and no electives… we also have to scale down the number of doctors and nurses that were around at any particular time” [P23, male, CMAC, urban tertiary facility, two years in role].

“We have a vibrant neonatal service, so we had a lot of premature babies that were in incubators, women still gave birth, they still had childhood problems, newborn issues, and prematurity issues so we kept on admitting and seeing patients” [ P13, female, MD, urban secondary facility, ten months in role].

“Now the immunisation clinic was never closed. It ran throughout the lockdown and is still running now” [P23 male, CMAC, urban tertiary facility, two years in role].

“We could not run any of the outpatient clinics. The gynae clinic, antenatal clinic, and the immunization clinic were affected. We opened the immunization clinic shortly. However, delivery and emergency services continued during that first month of April. From May, all services resumed except the gynaecological clinic, which did not open until about July” [P32 male, MD, urban secondary facility, one year and six months in role].

“You know before lockdown even when you come for clinic whether maternal, whether ante-natal clinic or child welfare clinic. We used to give them health talk but during lockdown no health talk. When they come in, we attend to them they leave. When they come in, we attend them, to them, they leave” [P4 female, Apex Nurse, urban primary health care facility, four years in role].

“Outreach services were affected, because people did not really want to come out and also, you know, limited transportation affected” [P16. female, Medical Officer of Health, urban primary health care facility, four years in role].

“Initially, General Hospital B was shut down because of the incidence of COVID-19 cases. This caused more work to us at PHC E. Patients were trooping in. Though we tried to maintain all the precautions… I mean even in the month of April, we had 82 deliveries, which is not normal” [P26, female, officer-in-charge, peri-urban primary health care facility, two years in role].


At the PHC level, though facilities remained opened, certain care components that required gathering of many patients like health education talks to pregnant women were discontinued. Attendance also reduced in many PHC outpatient clinics and outreach services, as many patients were concerned about the risk of infection in health facilities and others had difficulty in accessing transport (***Table 2***; P4, urban primary health care facility; P16, urban primary health care facility]. An officer-in-charge of a PHC close to a hospital that was shut down reported that the facility had to manage higher than usual number of patients during the hospital closure. (***Table 2***; P26, urban primary health care facility).

*Theme 2: Reorganizing service provision*

Long and staggered appointments were mostly used in secondary and tertiary facilities especially the ones in urban areas which typically had more patient numbers pre-pandemic. Health facilities took this approach as a way of minimizing number of patients visiting the facilities and achieving sufficient social distancing. Even for services like immunization that normally follow a schedule, a ‘catch-up immunization’ strategy was employed during this period for women who were unable to access this service during the lockdown. For example, mothers of some babies who were supposed to have received specific vaccines at six weeks were advised to come in at ten weeks and then the cycle continued (***Table 3***; P24, urban tertiary facility). In addition, the management introduced triage during which patients were screened at the port of entry to the hospital. In some instances, women were required to call the hospital line before commencing their journey to the hospital and a health worker would advise on the urgency or otherwise, and whether they need to travel to the hospital (***Table 3***; P18, urban tertiary facility). Hand washing, application of hand sanitizer at entry port, and use of facemasks were also mandated in many health facilities (***Table 3***; P14, urban secondary facility; P18, urban tertiary facility).

**Table 3:** Illustrative quotes for theme 2.


THEME 2 – REORGANIZING SERVICE PROVISION

“We tried to limit the number of people who were coming around to ensure that there was good social distancing. We staggered appointments and gave long appointment dates for non-emergencies” [P14, male, MD, urban secondary facility, three years in role].

“We did catch-up immunization. For example, those who are supposed to have received the vaccine at six weeks, some of them came at ten weeks and then we continued the cycle” [P24, male, HOD, urban tertiary facility, one year in role].

“The management introduced a kind of triage where people are screened before they even come into the hospital. They introduced the washing of hands, the use of hand sanitizer, the use of facemask” [P18, male, HOD PMTCT, urban tertiary facility, 17 years in role].

“For our nurses, we had to reschedule our roster so that we are not all exposed at the same time. We had some days that we don’t come and days we had to come. Throughout that period, there were nursing services available for every patient at the hospital” [P9, female, HOD Nursing, urban tertiary facility, three years in role].

“We adjusted our roster, so that our staff will come in and do 24 hours. They will then go home for four days. So that is how we were able to scale through that intense period of COVID-19” [P6, female, HOD Nursing, urban tertiary facility, four years in role].

“[To ensure sufficient social distancing], we shut down about half of the wards. So, what I mean is that we have four wards for older children and three wards for younger children. These three wards are divided into two, we have neonatal unit, and we have the post neonatal unit. During the lockdown, we shut down the post neonatal unit and collapsed the neonatal with post neonatal unit. Then the four wards were collapsed to just one ward, so most of the patients were managed in the emergency room and were discharged from the emergency room. Only those that needed follow up care were transferred to the wards” [P19, female, Director Clinical Training and Services, urban secondary facility, nine months in role].


Changes were also made to the working pattern of health workers while ensuring that service provision for inpatients was not compromised. The duty rosters were adjusted so that the health workers could work 24 hours at a stretch, then stay home for some days (***Table 3***; P6, urban tertiary facility). Facility leaders said this was done to reduce the risk of having health workers being constantly on the road, plying public transport, and getting exposed to the virus (***Table 3***; P9, urban tertiary facility].

Wards were also reorganized and, in some cases, shut completely. As much as was feasible, patients who visited secondary or tertiary facilities were managed in the emergency room and discharged from the emergency room without needing to be transferred to the wards (***Table 3***; P19, urban secondary facility).

*Theme 3: Leveraging technology for service provision*

Four secondary and tertiary facility leaders reported leveraging technology to support service provision during the initial phase of the pandemic. Health workers used WhatsApp forums to relate with mothers who required ANC, and for those who wanted to raise a concern about their pregnancy or the health of their children (***Table 4***; P22, rural secondary facility). One secondary hospital providing specialist maternity services also set up webinars and used its website to provide responses to frequently asked questions and advised patients to get information from their website (***Table 4***; P32, urban secondary facility). Direct phone calls to patients were also used in supporting care delivery in secondary and tertiary facilities located in both urban and rural parts of the State. This was mostly used for patients who only required follow up (***Table 4***; P32, urban secondary facility). Health facility leaders reported that there were some challenges with using technology to support care provision during the pandemic. This included inadequate and inaccurate patient record, inability to directly reach patients and the associated cost of purchasing and maintaining mobile phones for poor patients. (***Table 4***; P25, urban tertiary facility; P18, urban tertiary facility). Some patients also did not have smart phones, which are required for applications like WhatsApp (***Table 4***; P32, urban secondary facility).

**Table 4:** Illustrative quotes for theme 3.


THEME 3: LEVERAGING TECHNOLOGY FOR SERVICE PROVISION

“We went to the extent of having our own WhatsApp with our mothers in the ANC where staff related to mothers if they cannot come to the hospital. If they had any problem with their children, that’s a platform where they made their problem known and suggestions are giving there by doctors and nurses” [P22, female, Apex Nurse, rural secondary facility, four years in role].

“We had to refer some of the patients to our website, where they could get some information to guide them on their condition… on our WhatsApp group for our pregnant women, we continued to engage them with health talks, and we were also inviting them to some webinars” [P32, male, MD, urban secondary facility, one year and six months in role].

“We reached out to them by phone calls, which was done by several people and then I called somebody else called the same person, it was done and at least it achieved the goal of the calls because many of them responded” [P32, male, MD, urban tertiary facility, one year and six months in role].

“The pandemic exposed our weak health information management system. Our records are not electronic, most of them are paper-based and incomplete. Even when we tried to reach patients by phone, it was a challenge. Some was either a wrong number was given, or the numbers were not correctly inputted” [P25, female, HOD Paediatrics, urban tertiary facility, two years in role].

“Sometimes you know you call; they won’t pick it. Maybe it’s not theirs, it’s their husband’s phone number they put down or sometimes their own phone is not working, you try to call them it doesn’t go. Some went through and you’ll be able to encourage them to come especially when they’ve missed their clinic” [P18, male, HOD PMTCT, urban tertiary facility, 17 years in role].

“The only problem is that some of the patients were of low socio-economic level and so some of them did not have phones that can actually do some functions like WhatsApp. Their phones are the cheap ones that can only either call or text, which comes at a cost” [P32, male, MD, urban secondary facility, one year six months in role].


*Theme 4: Sourcing of resources for service provision*

The issue of inadequate PPE was a recurring challenge in many health facilities, especially the secondary and tertiary facilities. Many of the PHCs attested to rationing but not complete stock outs of PPE. Even when they were provided by government, some facilities raised concern with their quantity and quality (***Table 5***; P22, rural secondary facility).

**Table 5:** Illustrative quotes for theme 4.


THEME 4: SOURCING OF RESOURCES FOR SERVICE PROVISION

“PPE is not regular at all. I will be point blank and plain to you. They said they are coming to give us this PPE. I think they gave us five [Hazmat suits] (laughs)… Then they gave us sanitizer, liquid hand wash, nose mask. They found it difficult to give us and you know the hospital is not making much money. The PPE they gave us from central place from Lagos state, it wasn’t enough at all” [P22, female, Apex Nurse, rural secondary facility, four years in role].

“Because we didn’t keep quiet when they didn’t supply us. We began to fight them, that they must supply this PPEs by fire, by force. Maybe you [the leadership] will go and look for it, maybe you [the leadership] will go and buy it. I think with that we were able to scale through the risky period” [P20, female, Apex Nurse, urban secondary facility, 3 years in role].

“During the lockdown they [government] didn’t provide enough for us. The little one they bring we use it and when it finishes, we have to get it by ourselves. They didn’t use to supply us enough” [P21, female, CHO, rural PHC, four years in role].

“Well, we didn’t really have many challenges with PPE stock out. Since February, when we got a wind that the global pandemic started, basically, we doubled up on our supplies. Because usually what we get for a month, we ordered for two months. So, we bought ahead and had lots of supplies for routine work” [P32, male, MD, urban tertiary facility, one year and six months in role].

“Luckily for us people started donating these things [PPE] especially facemasks and surgical masks. We had some donors that gave us face shield… by that time things got improved” [P3, female, HOD Nursing, urban tertiary hospital, four years in role].

“We informed the executive chairman [of the Local Government] at that time and he provided some funds for facemask, gloves and other PPEs” [P15, male, MOH, rural PHC, one year in role].

“When we now had to restock, the prices of commodity had gone up. So, we even had to change some of our suppliers and do direct purchase because we know that there is a mark-up with them [suppliers]. So, we went into the market with our procurement committee to get some of the PPEs ourselves and that was able to save us some cost, even though it was still a bit higher than what we normally use to get” [P32, male, MD, urban secondary facility, one year and six months in role].


There was pressure on the leadership of secondary and tertiary health facilities to provide PPE. However, one facility reported having stocked adequate level of PPE even before the outbreak (***Table 5***; P32, urban secondary facility). During the pandemic, atypical sources of funding PPE were explored including use of personal funds at hospitals and PHCs, philanthropic personal donors, and some local government area (LGA) leaders (***Table 5***; P21, rural PHC; P20, urban secondary facility). PPE donations from non-governmental organisations (NGOs) and philanthropists were given mostly to secondary facilities especially those managing COVID-19 cases (***Table 5***; P3, urban tertiary facility) While LGA leaders were reported to have mostly supported PHCs (***Table 5***, P15, rural PHC).

For facilities who tried to source PPE themselves, a major challenge reported was the continuous price increase during this time. Some facilities had to change suppliers, while others engaged in open market purchase; others used direct purchasing to save cost (***Table 5***; P32, urban secondary facility).

*Theme 5: Building capacity of health workers for crisis*

Training, motivation, psychological support, and PPE provision for clinical and non-clinical health workers were used to build capacity of health workers to be able to safely maintain service delivery during the early phase of the pandemic (***Table 6***; P24, urban tertiary facility). This was in response to anxiety and concern about safety expressed by many health workers, especially those in secondary and tertiary facilities (***Table 6***; P32, urban secondary facility).

**Table 6:** Illustrative quotes for theme 5.


THEME 5: BUILDING CAPACITY OF HEALTH WORKERS FOR CRISIS

“Well, the first challenge was the anxiety, because there was a lot of anxiety and a lot of concern about their own safety. But through rigorous IPC training, generally reassuring them round and provision of PPEs, these kind of got them onboard” [P32, male, MD, urban secondary facility, one year six months in role].

“We have done training for all departments on infection prevention and control, from clinical staff to cleaners. Because it’s a chain, so we must… even if the cleaner is infected, they can infect everybody in the team. So, everybody had to be trained. And we have also provided the required PPE” [P24, male, HOD, urban tertiary facility, one year in role].

“Initially, they [health workers] thought that they had to wear the Hazmat suit for everything, so the COVID-19 response committee came up with what we called judicious use of PPEs. Knowing what PPE to use at what point in time, in terms of patient care. The training was well-taken up by members of staff. So that made it easier for us to stretch our PPEs” [Male, MD, urban secondary facility, one year and six months in role].

“So, we also had a guideline to assist the staff to know what level of PPE they will require for specific activities. These guidelines were pasted around the hospital at specific places for health workers” [P23, male, CMAC, urban tertiary facility, two years in role].

“We all started wearing scrubs, we all were meticulous about washing our hands, putting on our face mask, head gear, and goggles. We were doing that during lock down. Post lock down we are still doing that” [P19, female, DCST, urban secondary facility, nine months in role].

“What we did was to leverage our department of psychiatry partnering with an NGO and then set up a dedicated team that was ready to listen to staff on phone twenty-four seven (24/7) to provide psychological support to staff [of the facility]. This was satisfactory utilised by staff” [P23, male, CMAC, urban tertiary facility, two years in role].


With the pressure of limited PPE, the health workers were also trained on judicious and appropriate use of PPE (***Table 6***; P32, urban secondary facility). In addition, one tertiary facility led development of guidelines on use of PPE for specific activities (***Table 6***; P23, urban tertiary facility). Efforts were made to ensure that all staff were correctly and regularly wearing PPE and maintaining a high level of IPC during and after lockdown (***Table 6***; P19, urban secondary facility).

One hospital specifically mentioned that they provided round-the-clock psychological support to health workers with support from in-house psychiatry consultants and non-governmental organization (NGO) (***Table 6***; P32, urban tertiary facility).

## Discussion

### Summary of key findings

Our study highlights several unusual decisions made, actions taken, and adaptations implemented by health facility leaders across different tiers of public health service in Lagos state with a goal of optimizing health service provision during the early phase of the COVID-19 pandemic. Within the context of the unparalleled disruption and crisis that the COVID-19 pandemic had placed on the health system, health facility leaders worked with their teams, explored new partners, and received guidance from government to institute solutions and adaptations for service provision as new policies for slowing down the spread of the pandemic emerged. Five broad adaptations were recounted by facility leaders: scaling down and discontinuing some MNCH services, reorganizing MNCH service provision, leveraging technology for service provision, building all-round capacity of health workers for the crisis, and exploring new ways of sourcing resources for service provision.

### Interpretation of findings

In our study, many public hospitals discontinued some outpatient MNCH services and elective surgeries in Lagos. In a global survey conducted between March 24 and April 10, 2020, this response was similarly reported by health workers in many countries, irrespective of income level [6]. However, in Lagos, our study shows that critical services like immunization were continued in several facilities throughout the lockdown, although uptake was suboptimal. This is in contrast to alarming disruptions or delays in routine immunization reported in many other LMICs [23]. A crucial point that our study highlights is that the disruption caused by the pandemic was nuanced. While hospitals that took care of many of the sicker persons scaled down services, many PHCs remained open, though service utilization by women was still low because of the fear of nosocomial infections. There were also collateral effects of service closures. For example, in our study, scale down of services in hospitals meant that some PHCs had to manage more patients. The rationale for having to scale down services during the initial phase of the pandemic was clear – reduce the spread of the disease – however, women and children still had health needs, and these still needed to be addressed, warranting alternative arrangements. Similarly, in Canada, women were offered the option of home birth during the early part of the COVID-19 pandemic [6].

To minimise risk of infection, service reorganization that involved triaging, mandatory hand sanitising, wearing of facemasks, changing to 24-hour duty shift patterns, and restructuring of physical ward spaces were implemented across different levels of care in Lagos. Like the discontinuation of services, the rationale for these changes were clear. For triaging, some women were required to call the hospital ahead to assess whether they needed to travel to the hospital or not. This was not a practice that was routinely conducted before the pandemic and many obstetricians queried whether it was feasible in Nigeria, despite it being recommended by their professional organization [24]. That this was introduced is commendable and it comes with supply benefits, including improved efficiency and performance of health workers. For demand, it can lead to reduced delay for women after they arrive at the facility. These are some of the gains which were reported in a study conducted in Ghana which concluded that if properly implemented, an obstetric triage system can work in an LMIC setting [25]. However, the concern that many women who need care in Nigeria do not have access to mobile phones to be able to ring in for an obstetric triage [24], is indeed valid.

In our study, leaders of some secondary and tertiary facilities reported leveraging technology for service delivery by using WhatsApp and direct phone calls to connect with patients. In a global survey conducted for health workers rendering maternity care during the early phases of COVID-19, about 58% of them resorted to one or more telemedicine tools in engaging their clients [26]. This was a critical strategy to maintain connection with patients, as over a third of women in Lagos could not access reproductive, maternal, newborn, and child health (RMNCH) services as a result of the lockdown and about a fifth said they could not access transportation to reach health facilities during the early parts of the pandemic [27]. Questions have long been asked regarding the possible role of telemedicine in LMICs. Although the need to explore more innovative means to engage with patients came with the pandemic, the challenges reported in our study – including inaccurate capture of patient information and cost to patients for buying, maintaining, and servicing mobile phones to access the service – remain issues that need to be considered.

Training, motivation, psychological support, and PPE provision were some of the approaches used by health facility leaders to build capacity and resilience of health workers for the crisis period. For the trainings, many were done in conjunction with the Ministry of Health and donor agencies. In a survey conducted during the same period as our study, almost three-quarters of health workers received COVID-19 training in Lagos. Regarding PPE, health facility leaders in our study talked about the health workers themselves needing training on appropriate use of PPE to avoid wastage. It is well established that demand for PPE outstripped supply at the beginning of the pandemic; 97% of RMNCH workers in another Lagos study were concerned about sufficiency of PPE [12]. Indeed, the shortage was so high that even women requiring service had to pay significantly huge sums for them on top of their care cost [28]. Health facility leaders made efforts to explore innovative and “unusual” approaches such as direct purchasing, purchases from open market, advocacy to politicians and philanthropists to increase PPE supplies. Our study brings to the fore, the need for better emergency preparedness especially for commodities and supplies to ameliorate the cost-of-service provision and availability of PPE for health service delivery. Only one health facility leader could point to a robust strategy for stockpiling commodities ahead of the pandemic.

#### Strengths and limitations

To the best of our knowledge, this is one of the first studies that holistically documents actions and adaptations implemented by health facility leaders in a part of sub-Saharan Africa, to support MNCH service provision during the COVID-19 pandemic. Within our sample, there was a good mix of leaders including nurses/midwives and medical doctors with varying years of experience. Indeed, our sample frame included all public-owned facilities in Lagos state and our findings are representative of perspectives across the entire three-tier public health system in Lagos state. Our sample was also reflective of the preponderance of female frontline health workers for MNCH services in Nigeria [29]. However, as a limitation, we have not collected any data from the perspective of the private sector providers in Lagos state. We have a case for focusing on the public sector, as four and two times the number of women in Nigeria receive antenatal care and childbirth service in public hospitals respectively compared to private hospitals according to the most recent Nigeria Demographic Health Survey [17,30].

### Implications for policy, practice, and research

Health facility leaders in Lagos implemented diverse change initiatives during the COVID-19 pandemic. Faced with diverse pressures, incapacitation of facility leaders to be able to lead during a crisis like the ongoing COVID-19 pandemic can result in significant consequences for patients, skilled health personnel and the leaders themselves [31]. Ensuring that they are provided with sufficient support and guidance about the crisis while being given the latitude to respond to bespoke challenges is essential for maintaining service provision in such situations. While there are presently no studies assessing the effectiveness of the approaches implemented during the crisis, there is a need to ask stakeholders if they worked, why they worked if they did, and how they can be better implemented.

## Conclusion

COVID-19 was certainly a catalyst for service change [32], and health facility leaders were not only at the forefront of the frontline of the COVID-19 response but also leading the implementation of changes to maintain service provision. While they took to implementing sensible guidelines from global, national, and state levels within their facilities, they were also driven to be innovative in their response to the indirect effects of COVID-19. A compendium of actions taken like we have shown in our study will be crucial lessons to foster resilient health systems if indeed COVID-19 becomes our “new normal” or MNCH service provision become compromised in the future during another crisis.
